# Design and analysis of mismatch probes for long oligonucleotide microarrays

**DOI:** 10.1186/1471-2164-9-491

**Published:** 2008-10-17

**Authors:** Ye Deng, Zhili He, Joy D Van Nostrand, Jizhong Zhou

**Affiliations:** 1 Institute for Environmental Genomics, Department of Botany and Microbiology, the University of Oklahoma, Norman, OK 73019, USA

## Abstract

**Background:**

Nonspecific hybridization is currently a major concern with microarray technology. One of most effective approaches to estimating nonspecific hybridizations in oligonucleotide microarrays is the utilization of mismatch probes; however, this approach has not been used for longer oligonucleotide probes.

**Results:**

Here, an oligonucleotide microarray was constructed to evaluate and optimize parameters for 50-mer mismatch probe design. A perfect match (PM) and 28 mismatch (MM) probes were designed for each of ten target genes selected from three microorganisms. The microarrays were hybridized with synthesized complementary oligonucleotide targets at different temperatures (e.g., 42, 45 and 50°C). In general, the probes with evenly distributed mismatches were more distinguishable than those with randomly distributed mismatches. MM probes with 3, 4 and 5 mismatched nucleotides were differentiated for 50-mer oligonucleotide probes hybridized at 50, 45 and 42°C, respectively. Based on the experimental data generated from this study, a modified positional dependent nearest neighbor (MPDNN) model was constructed to adjust the thermodynamic parameters of matched and mismatched dimer nucleotides in the microarray environment. The MM probes with four flexible positional mismatches were designed using the newly established MPDNN model and the experimental results demonstrated that the redesigned MM probes could yield more consistent hybridizations.

**Conclusion:**

This study provides guidance on the design of MM probes for long oligonucleotides (e.g., 50 mers). The novel MPDNN model has improved the consistency for long MM probes, and this modeling method can potentially be used for the prediction of oligonucleotide microarray hybridizations.

## Background

DNA microarray technology has been widely used in gene expression analysis, disease characterization, environmental monitoring and many other biological processes. PCR amplicons [1], oligonucleotides [2] and genomic DNA [3] have all been used as microarray probes. Currently, the use of oligonucleotide probes has become popular due to better specificity, easier construction, and less cost [4,5] compared to other probe types. In addition, many studies [4,6,7] have demonstrated that longer oligonucleotide probes (e.g., 50 mers or longer) yield better sensitivity than shorter probes (e.g., 20–30 mers). Therefore, long (e.g., 50-mer, 70-mer) oligonucleotide probes have been widely used, especially for spotted oligonucleotide microarrays.

Nonspecific hybridization is one of the major concerns with microarray technology. This is usually caused by highly homologous sequences, such as gene families or multi-spliced variants [8,9]. Owing to the high similarity with target sequences, false positive signals contribute to the total signal intensities and they are difficult to subtract out. Results from Kane et al. [10] suggest that, for long oligonucleotides, a probe sharing >75% identity with non-targets might cause significant cross-hybridization. Further experiments showed that the oligonucleotide specificity was also affected by high local sequence similarity (identical stretch length), free energy and other factors [11-13]. Although deciphering the source of nonspecific signals can be quite complicated, a simple experimental option to handle nonspecific signals is to design one or more pairs of perfect match (PM) and mismatch (MM) probes for each gene [4,7,14]. The signal from the MM probe would then represent nonspecific cross-hybridization to the corresponding PM probe. Hence, subtracting the signal intensity of the MM probe from its PM probe would theoretically eliminate the nonspecific signal and would be directly proportional to the concentration of the real target [2,15]. Currently, PM/MM pairwise probes have been widely applied in short oligonucleotide microarrays. For example, the Affymetrix GeneChip^® ^usually employs 11 different 25-mer PM/MM probe pairs for each gene [14]. Many statistical methods have been developed to estimate the binding affinity of probe-target duplexes on this platform [16-19]. One of the most commonly used methods is the positional-dependent-nearest-neighbor (PDNN) model [20,21]. This model is based on the fact that different positions of a probe may contribute differently to the binding affinity [22,23], so that the binding free energy of a probe could be expressed as a weighted sum of its nearest-neighbor (NN) stacking energies [21]. Although all of these methods are effective in correcting some background noise in Affymetrix^® ^microarrays, it is still difficult to explain the observation that up to a third of MM probes had higher signals than their PM probes [24]. Although efforts have been taken to explain and solve this problem by altering the physical and chemical characteristics of the chips [25-28], the uncertainty of MM probe signals may be caused by the design of the MM probe. To create each MM probe, a single nucleotide is replaced at the middle position of its corresponding PM probe [14]. This arbitrary design has not been experimentally validated and hence the signals of MM probes are difficult to predict accurately. As a result, higher signal intensities obtained from the MM probes compared to their PM partners were usually considered to be from bad probe pairs and were completely removed or ignored [29]. Therefore, in order to accurately predict the signal intensities for oligonucleotide probes in microarray hybridizations, a more elaborate design and testing of MM probes seems necessary.

A few studies have been conducted with MM probes on chips [2,14], but this approach has not been used for long oligonucleotides. Further, specific detection of unknown targets, such as environmental samples, requires MM probes to eliminate the influence of nonspecific hybridizations for long oligonucleotide arrays. In this study, we used a microarray with 50-mer PM and MM probes to determine the parameters for MM probe design. Our results demonstrated that evenly distributed MM probes with three to five mismatched nucleotides were suitable for 50-mer oligonucleotide probes under different hybridization conditions. A modified positional dependent nearest neighbor (MPDNN) model was then established for a better prediction of hybridization signals for long oligonucleotide microarrays. This study provides general guidance for long oligonucleotide probe design.

## Methods

### Oligonucleotide probe design and microarray construction

Ten genes from *Desulfovibrio vulgaris *Hildenborough, *Shewanella oneidensis *MR-1 and *Methanococcus maripaludis *were randomly selected as target sequences (Table 1). The best 50-mer probe was designed for each gene using the CommOligo program [30] with parameters based on optimized experiments [11]. Seven groups of MM probes were designed for each PM probe with 1–7 mismatch nucleotides, respectively. For the nucleotide exchanges, Gs or Cs in the PM probes were changed to As, and As or Ts were changed to Gs. Each MM group consisted of two kinds of MM probes: one with evenly distributed mismatches and three with randomly distributed mismatches. For evenly distributed MM probes, the interval between two mismatches or between mismatch and terminus was as long as possible. For example, three mismatches were located at the 12th, 25th and 38th positions of the probe strings and four mismatches were located at the 10th, 20th, 30th and 40th positions. After all probes were designed, they were searched against the GenBank database. There were no highly similar hits or SNPs found with these PM and MM probes. In total, 290 probes, including 10 PM probes and 280 MM probes (Additional file 1) were commercially synthesized at a concentration of 100 *μ*M (Invitrogen Life Technologies, Carlsbad, CA). All probes were prepared in 50% dimethyl sulfoxide (Sigma-Aldrich, St. Louis, MO) and spotted onto UltraGAPS slides (Corning Inc., Corning, NY) using a MicroGrid robotic arrayer (Genomic Solutions, Ann Arbor, MI). Each probe was printed four times on each slide (1160 spots per array). After printing, the long oligonucleotide probes were covalently fixed to the surface of the slide by cross-linking at 600 mJ (UV Stratalinker 2400, STRATAGENE, La Jolla, CA) and then stored at room temperature.

**Table 1:** Genes used for perfect match (PM) and mismatch (MM) probes.

**ID**	**Genbank ID**	Organism*	**Annotation**
DVU0625	46451220	DvH	Putative cytochrome c nitrite reductase, catalytic subunit NfrA
DVU1466	46451220	DvH	Acetylglutamate kinase (argB)
DVU1782	46451220	DvH	Iron-sulfur cluster-binding protein
DVU2526	46451220	DvH	Periplasmic [NiFe] hydrogenase, large subunit, isozyme2(hynA-2)
MMP0707	44921025	Mm	Na+/H+ exchanger
MMP0926	44921025	Mm	Chemotaxis protein cheB
MMP1559	45047480	Mm	Formatedehydrogenase alpha subunit
SO1362	24371479	So	Chorismate mutase/prephenate dehydrogenase (tyrA)
SO1779	24371479	So	Decaheme cytochrome c (omcA)
SO2452	24371479	So	Alcohol dehydrogenase, zinc-containing

*DvH: *Desulfovibrio vulgaris *str.*Hildenborough*; Mm: *Methanococcus maripaludis*; So:*Shewanella oneidensis *MR-1.

### Microarray hybridization and data processing

The synthesized oligonucleotides were prepared in the same manner as the artificial targets. For each PM probe, a complementary 50-mer oligonucleotide target was commercially synthesized with a Cy3 or Cy5 fluorescent dye (MWG-Biotech, Ebersberg, Germany) at the 5'-end. Hybridizations were performed using a Tecan HS4800 Hybridization Station (Tecan US, Durham, NC). Hybridizations were carried out as described previously [31]. Briefly, hybridization solution [130 *μ*L; 50% formamide (Mallinckrodt Baker, Phillipsburg, NJ), 3× saline-sodium citrate (SSC), 0.3% SDS, 0.8 mM DTT, 0.7 *μ*g/*μ*L of herring sperm DNA (Invitrogen Life Technologies, Carlsbad, CA)] was mixed with the labeled targets, heated to 98°C for 5 min, and then kept at 65°C until ready for injection. After the injection of hybridization solution, the hybridization was carried out at 42, 45 or 50°C for 10 hours with agitation. Microarrays were scanned with a ProScanAarray microarray Scanner (Perkin-Elmer, Boston, MA) at 90% laser power and 80% photomultiplier tube efficiency [32,33]. Hybridizations were conducted in duplicate for determining the optimal target concentration and in triplicate for all other experiments.

The images obtained from scanning were analyzed by using ImaGene 6.1 (Biodiscovery Inc., El Segundo, CA). The ambient background for each spot was measured independently. All pixels within approximately a half radius of each spot were used to calculate the background mean and standard deviation of the spot. The signal-to-noise ratio [SNR; SNR = (signal mean - background mean)/(background standard deviation)] was then calculated for each spot to discriminate true signals from noise. Spots with an SNR equal to or greater than 2.0 were considered positive [34]. Probes for which more than 50% of the total numbers of spots were positive were regarded as valid probes. Both valid and invalid probes were used to calculate the average signal in the primary computations, but only valid probes were used to construct the following model.

### Modified Positional Dependent Nearest Neighbor model

The PDNN model can be expressed as [21]:

(1) $$\Delta G=\sum_{k=1}^{n-1}\omega_{k}\varepsilon (b_{k},b_{k+1})$$

where Δ*G *is the free energy of a target-probe duplex; *n *is the number of probe length; *ω*_*k *_is a weight factor that depends on the position *k *along the probe; and *ε *(*b*_*k*_, *b*_*k*+1_) represents a stacking energy term [35,36].

Except for this key formula, other mathematical regressions in the PDNN model are based on the multiple pairwise PM/MM probe sets of GeneChip^® ^microarrays. Herein, we modified the PDNN model to fit 50-mer oligonucleotide arrays with a single pair of PM/MM probes and to acquire more precise thermodynamic parameters for the spotted microarray platform. Two major modifications were made. First, the assigned weights for the PM probes in the PDNN model gave the highest weight to the middle position with decreasing weights on either side until reaching the two fraying ends. Based on a recent study of microarray thermodynamics [16], the MM probes were assigned mismatch penalties for the binding free energy at mismatches and the two adjacent nucleotide positions. Second, we adopted a simple linear relationship between relative signal intensity and relative free energy to perform a mathematical regression:

*S*_*MM*_/*S*_*PM *_∝ Δ*G*_*MM*_/Δ*G*_*PM*_

where *S *and Δ*G *represent the signal intensity and free energy, respectively, and *S*_*MM*_/*S*_*PM*_, Δ*G*_*MM*_/Δ*G*_*PM *_are the relative signal intensity and relative free energy of the MM probe for its corresponding PM probe.

In the MPDNN calculation, temperatures were set to 30°C higher than the actual hybridization temperatures because the hybridization buffer contains 50% formamide which is expected to destabilize duplexes in a way equivalent to increasing the temperature by this amount (0.6°C per 1% formamide) [37]. All calculation programs were written in PERL script and run in the Windows environment.

## Results

### Distribution of mismatch positions

One important parameter for MM probe design is the distribution of mismatch nucleotides within the probe string. To investigate the effect of mismatch position on signal intensity, the 50-mer artificial oligonucleotide probes with 1 to 7 mismatches were used. Ten synthesized targets complementary to the 10 PM probes were mixed equally in different concentrations to determine the optimal concentration for the spotted microarrays at 45°C. The experimental results showed that at all concentrations tested the signal intensities were lower for MM probes with higher numbers of mismatches. These trends were consistent with those from different concentrations of targets (data not shown). From these data, 10 pg of synthetically labeled target, equivalent to 30 fM per target, was needed to achieve appropriate specificity and sensitivity for the constructed microarrays. Therefore, this experimentally determined optimal concentration of target was used for the following studies.

**Figure 1:**
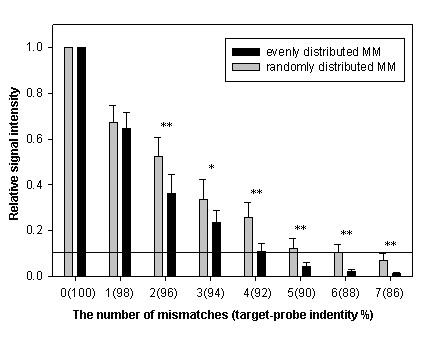
**Comparison of the relative signals between evenly- and randomly-distributed MM probes.** The x-axis is the number of mismatches in the probes. The value in brackets is the percent identity of the 50 bp probe-target duplex. The y-axis is the average relative signal intensity of ten genes. Error bars indicate standard error of replicate arrays. Statistical tests were done between evenly and randomly distributed probes. An asterisk (*) indicates p-values ≤ 0.05, and double asterisks (**) indicate p-values ≤ 0.01 using a paired T-test.

The influence of mismatch position distribution was assessed through changes in the relative signal of MM probes to their corresponding PM probes (Figure 1). Approximately 70% of PM signals were detected for single mismatch probes, and no significant differences in relative signal intensities were observed between randomly and evenly distributed probes (Figure 1). However, the effect of mismatch position on signal intensity was obvious for two or more mismatches with 36% and 52% for evenly and randomly distributed two mismatches, respectively, 23% and 33% for three mismatches, 11% and 26% for four mismatches, 4% and 12% for five mismatches, 2% and 11% for six mismatches, and 1% and 9% for seven mismatches (Figure 1). Although an increase in the number of mismatches markedly reduced the relative signals for both evenly and randomly distributed MM probes, the data also showed that the relative signals of evenly distributed MM probes decreased faster than those of randomly distributed MM probes when the number of mismatch nucleotides was the same. For example, probes with four mismatches with a random distribution had an average of 31% PM signal compared to 13% for evenly distribution (Figure 1). The Student T-test results revealed that the differences between evenly and randomly distributed MM probes became significant when the number of mismatches was equal to or greater than two. These results indicate that the positional distribution of mismatches greatly affects the performance of MM probes on microarrays, and that an even distribution may make it easier to distinguish MM probes from their corresponding PM probe, suggesting that an even distribution is better for MM probe design.

### Determination of the numbers of mismatches at different hybridization temperatures

**Figure 2:**
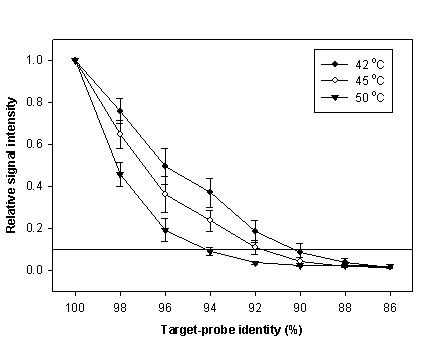
**The relationship between probe-target identity and relative signal intensity at different hybridization temperatures.** Each data point is the mean value from ten evenly-distributed MM probes. Error bars indicate standard error of replicate arrays.

To evaluate the effect of temperature on the hybridization the signal intensity of PM/MM probe pairs and to further determine the optimal number of mismatches for 50-mer MM probes, the microarrays were hybridized at 42, 45 or 50°C in the presence of 50% formamide, which are the most commonly used hybridization conditions for spotted microarrays. As shown in Figure 2, the relative signal intensities decreased as the number of mismatch nucleotides and the temperature increased. For instance, at 42°C, the MM probes with two and five mismatches had about 50% and 20% of the PM signal, respectively, but only about 38% and 10% at 45°C, and less than 20% and 5% at 50°C (Figure 2). With five mismatches, the relative signal intensities were all less than 0.1 (0.087 at 42°C, 0.044 at 45°C and 0.025 at 50°C). If a relative signal intensity of ≤ 0.1 is considered background noise [11], three evenly distributed mismatches were needed for MM probes hybridized at 50°C, four were needed at 45°C and five mismatches were required at 42°C.

### MPDNN modeling

Although the average relative signal intensity generally decreased with decrease in probe-target identity under all temperatures examined, the relative signal variation among the ten currently designed PM/MM probe pairs was still considerable. For instance, ten evenly-distributed MM probes with four mismatches had relative signal intensities from 0.006 to 0.62 with a standard deviation of 0.23. We considered this variation to be too large to properly evaluate non-specific hybridization in the microarray system. Therefore, an appropriate model had to be developed in order to design MM probes with more accurate predictions. Here we present key steps used to modify and improve the PDNN model [20,21].

**Figure 3:**
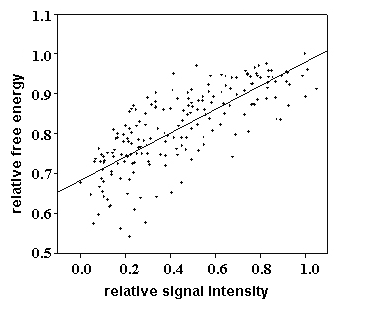
**The linear relationship between relative signal intensity and relative free energy.** Here, the original free energy of each probe was calculated by the Nearest Neighbor method.

First, considering the specific and nonspecific signals that are primarily determined by binding affinities of the DNA duplex, and that affinity is determined by free energy thermodynamics, we calculated the correlation between relative signal and relative free energy. Because weak spots are normally too ambiguous to reflect real hybridization behavior, only spots with SNR >2.0 using 10 pg of each synthetic target at 45°C were chosen as valid data. The stacking energies of dimer nucleotides from the NN method [9,35,38-40] were initially used to calculate the free energy of each probe. The relative signal intensities and original relative free energies of valid data were plotted in Figure 3. Generally, the relative signals increased with an increase in relative free energy. The regression analyses indicated a linear correlation had a higher relative coefficient (R = 0.798) than logarithmic (R = 0.736) or power (R = 0.741) correlations. Therefore, a linear correlation between relative signals and relative free energies was used for model construction.

**Figure 4:**
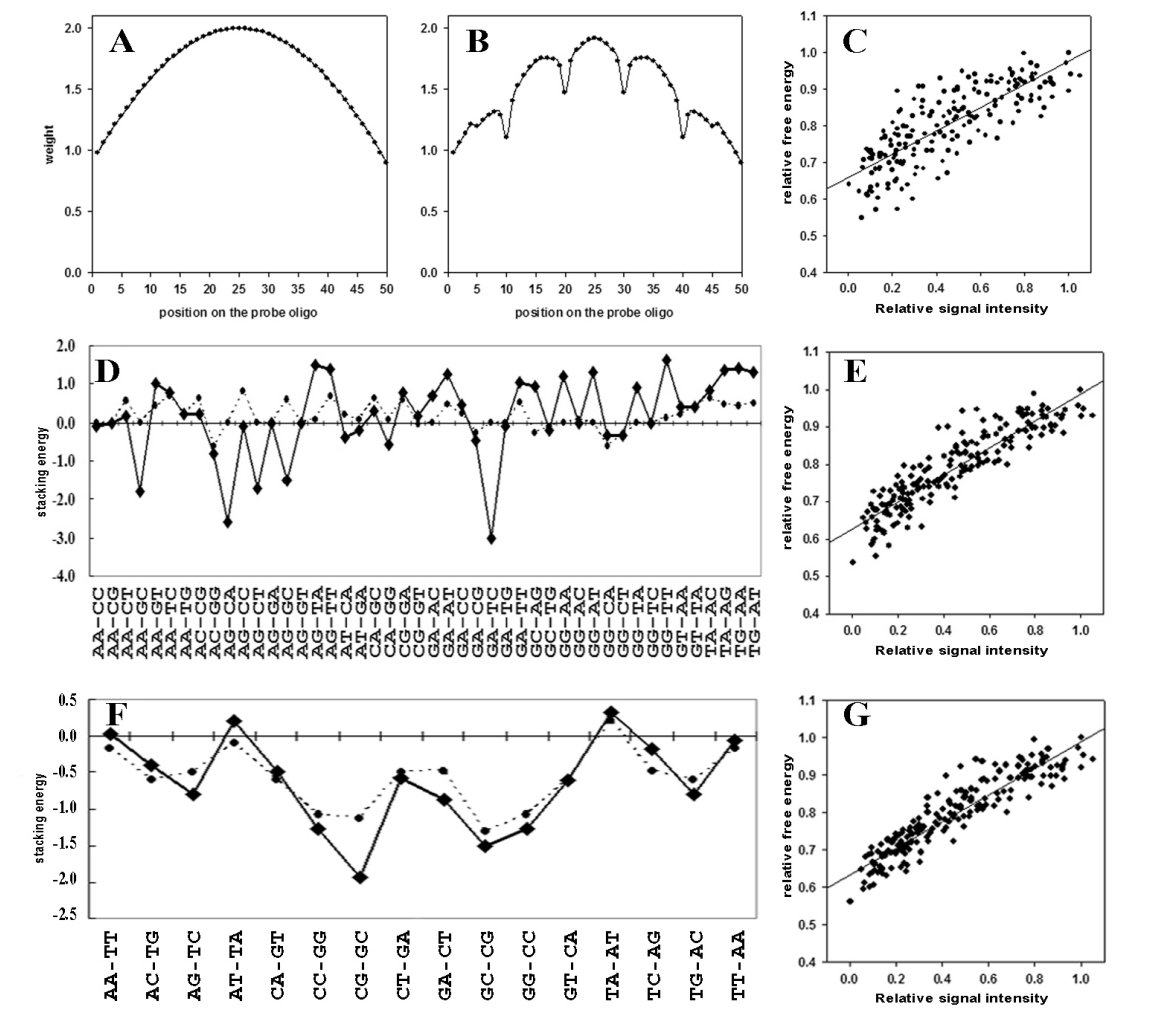
**The linear regression of relative signals with relative free energies in the MPDNN method.** A. Weight factors and positions of 50-mer PM probes in the MPDNN model. B. Weight factors of a MM probe with four evenly distributed mismatches. C. Linear scatter plot of relative signals and relative free energies after assigning positional weights. D. Parameters of MM dimer nucleotides in the NN and MPDNN models. The dotted line is the parameter based on a 75°C water solution based on calculations for the NN method. The solid line is the parameter obtained from the MPDNN model. E. Scatter plot of relative signals with relative free energies after the parameters of the dimer MM nucleotides were adjusted. F. The parameters of PM dimer nucleotides in the NN and MPDNN model. The dotted line is the parameter from the NN method and the solid line is the parameter observed from the MPDNN model. G. Scatter plots of relative signals with relative free energies after the parameters of dimer PM nucleotides were adjusted.

Second, a weight was assigned for each probe position. The distribution of weight factors along the probe was adopted from the PDNN model for 50-mer probes. For PM probes, the weight distribution was similar to a normal distribution, in the range of one delta (Figure 4A). For MM probes, considering that mismatched nucleotide pairs form a bubble in the DNA string, which would affect several adjacent nucleotides, positional weights were assigned by reducing the weight of mismatched positions and five of the adjacent positions. For example, the weights of probes with four evenly distributed mismatches are shown in Figure 4B. The thermodynamic parameters for solutions were used in calculating free energies. After assigning these new positional weights, the correlation was considerably improved to 0.828 (Figure 4C).

Third, each thermodynamic parameter for the mismatched dimer nucleotides was amended to fit the best linear correlation of relative signals and relative free energies. The original and optimized parameters (Figure 4D) used increased the correlation value of the obtained scatter plots to 0.921 (Figure 4E). Some parameters of mismatch dimer nucleotides in the NN method were substantially adjusted in our modified PDNN (MPDNN) model, for example, AG-CA which denoted a double mismatches pair (A-C and G-A) was decreased from 0 to -2.1, and AG-CC was decreased from 0.8 to -0.1. Further, the correlation coefficient increased from 0.828 to 0.921, indicating that these parameters significantly affected the linear relationship between the relative free energy and the relative signal intensity.

Finally, each thermodynamic parameter of matched dimer nucleotides was adjusted (Figure 4F), and the related coefficient reached 0.938 (Figure 4G). An obvious result was that several equivalent parameters in solution became inconsistent with those in the spotted microarray system. For instance, due to the symmetry of the DNA duplex, the thermodynamic parameters of AG-TC and TC-AG were both -0.49 in solution, but from the microarray data we obtained thermodynamic parameters of -0.80 and -0.40, respectively. This suggests that the DNA duplexes are asymmetric on the microarray surface, and that the probes and targets contribute differently to the thermal stability of their complexes.

### Redesigned MM probes through MPDNN

**Figure 5:**
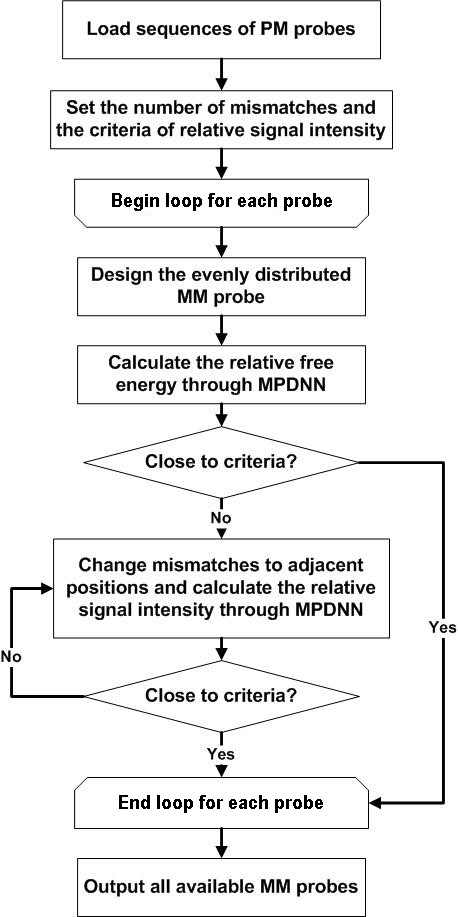
Flowchart of MM probe design with the novel MPDNN model.

The MPDNN model and the modified thermodynamic parameters of dimer nucleotides were used to identify MM probes. It was expected that the calculations of the relative free energy using MPDNN modified parameters could better predict the relative signal intensity for each MM/PM probe pair. The basic process of MM probe design is shown in Figure 5. Each PM probe was designed with evenly distributed MM probes first, and then the relative free energy was calculated through the MPDNN model. If the obtained value was significantly different from the predetermined criterion of relative signal intensity (0.1 in this study), the program would replace each nucleotide adjacent to the mismatched nucleotides in the original MM probe to obtain a new MM probe. The relative free energy was then recalculated until a best MM probe was identified so that the relative free energy was closest to the criterion. The newly designed MM probes have relatively flexible positioning of mismatches instead of the fixed mismatch positions in the previously designed MM probes.

**Figure 6:**
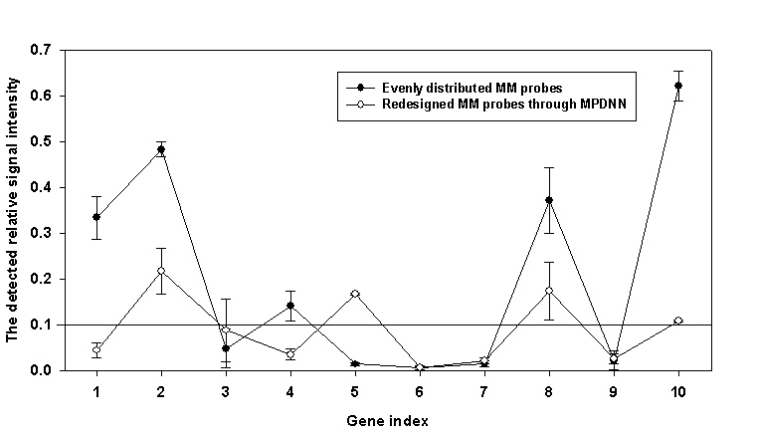
Comparison of experimentally detected relative signal intensities between evenly-distributed and flexible positional MM probes through the MPDNN model.

To test if the MPDNN model could reduce variation among different MM/PM pairs and improve prediction of probe hybridization, ten probes with four mismatched nucleotides were redesigned using the MPDNN model with the criterion of relative signal intensity set to 0.1 (Table 2) and commercially synthesized. These newly redesigned probes were printed on the same microarray with all other previously designed probes and hybridized with the complementary oligonucleotides. The experimental results showed that all high relative signal intensities (>0.3) from the previously designed MM probes were successfully decreased to less than 0.21 using the newly designed MM probes with flexible positional mismatches. The standard deviation was 0.07 for the ten redesigned MM probes, much less than the 0.23 obtained in the previous MM probes with fixed positional mismatches (Figure 6). These results demonstrate that all ten relative signal intensities were much closer to the criterion of 0.1 and that the consistency of relative signal intensities for all MM/PM probe pairs was significantly improved.

**Table 2:** Comparison of the evenly-distributed MM probes and the MM probes designed using the MPDNN modeling parameter

**Name**	**Type**	Sequence*	RSI**
DVU0625_MM4	Evenly	GCAGGCTATAACGACCTGAGGATCCAGGCACGTGAGATGATCCGCAAGGG	0.345
	MPDNN	GCAGGCTATAACGACCTGATAATCCAGACCCGTGAGATAGTCCGCAAGGG	0.109
DVU1466_MM4	Evenly	TGGCAAGGTAGGCGAAGTGGTGGGCGTGAGCACGACGCTGCTGCGTTCTC	0.527
	MPDNN	TGGCAAGGTGGACGAAGTGATAGGCGTAAACACGACGCGACTGCGTTCTC	0.158
DVU1782_MM4	Evenly	GGGTGGGAGGTGGTCTACAACCATCCTGCACTGTATTCCGTCGTCTTGAA	0.190
	MPDNN	GGGTGGGAGAGGGTCTACAACCATCCTGCCCGGTATTCATTCGTCTTGAA	0.100
DVU2526_MM4	Evenly	AAGGTCGAGAAGGTGAACCAGGAACAGATGGTGGAGCATATGGCCCACAG	0.345
	MPDNN	AAGGTCGAGGAAGTGAACCCAGAACAGATAATGGAGCATGTAGCCCACAG	0.136
MMP0707_MM4	Evenly	CAGAGGAGTGGTTCCTGCGACACTTGCGGAAATGATATAAACAAATATTA	0
	MPDNN	CAGAGGAATAGTTCCTGCGACACTTGCGGCAGTGATATGCACAAATATTA	0.100
MMP0926_MM4	Evenly	ATTAAACAGATTAAAGATGATTCAAAATCAAAAGTAAGAGTTAAATCATC	0
	MPDNN	ATTAAACAGGGTAAAGATGATTCAAAATCCGAAGTAAGAAGTAAATCATC	0.042
MMP1559_MM4	Evenly	ATTAAAAGCGGCAATTGGTGAAAAAACATGCCAAGTATCGAGAGTTCCAT	0
	MPDNN	ATTAAAAGATGCAATTGGGAAAAAAACATGCCAAGTATCGAGAGTTCCAT	0.056
SO1362_MM4	Evenly	GGTAGTTATGGTGGGCGGTGAAGGCCAGCGTGGCGGGCTATTTCAACAAA	0.353
	MPDNN	GGTAGTTATAATGGGCGGTAAGGGCCAACTTGGCGGGCGGTTTCAACAAA	0.102
SO1779_MM4	Evenly	CGCATTTCGGTTGGCAACCGTCAACAGGTGAAACAGAAGACATTCAAACT	0
	MPDNN	CGCATTTCGGTTGGCAAACTTCAACAGGTGAAACAGAAAGCATTCAAACT	0.101
SO2452_MM4	Evenly	GTTACATGAATTTGGTGATAGTCAAGATTAGCAGATCCTAATGCAACAAG	0
	MPDNN	GTTACATGACTGTGGTGGTCGTCAAGAGTGGCAGATCCTAATGCAACAAG	0.101

*The 50-mer MM probe sequences. The letters in shadow are the mismatched nucleotides.

**The predicted relative signal intensity (RSI) using the MPDNN model.

## Discussion

One of the biggest challenges for microarray-based approaches is to eliminate non-specific hybridization so that probe signals accurately reflect the presence and concentration of specific targets. Besides several commonly used methods to increase hybridization specificity in experimental steps, such as increasing the hybridization temperature, the PM/MM probe pairwise design is one of the most accurate approaches to minimize or eliminate the effects of cross-hybridization. Most microarray manufacturers have not thought it necessary to use MM probes for long oligonucleotide arrays since these probes have a higher specificity than shorter probes. Additionally, inclusion of MM probes would double the cost and reduce the capacity of the microarrays. However, in practice non-specificity is a big issue for long oligonucleotide arrays, especially for complex samples, like environmental DNA, which would have many unknown sequences, some of which could be similar to those PM probes. Furthermore, with the continuing development of microarray technology, the capacity of microarrays is expected to increase. In this study, we applied this pairwise approach to long oligonucleotide (50-mer) probe design and experimentally established MM probe design parameters for the number and position of mismatches. In addition, instead of simply altering the nucleotides at fixed positions within the probe string, we constructed a model to calculate the relative free energy based on microarray data, and then used this model to further improve MM probe design. The results demonstrate that this novel MPDNN model can provide guidance for long (50-mer) PM/MM oligonucleotide probe design.

Currently, there are no commonly accepted methods for MM probe design, especially for long MM oligonucleotides. The simplest method, used by Affymetrix GeneChip^®^, replaces the central nucleotide of a 25-mer PM probe. However, this caused an unexpected consequence of higher signal intensity for up to a third of MM probes compared to their PM probes [24]. Therefore, to obtain a useful MM probe, two issues should be considered. First, the MM probe must be as similar to its corresponding PM probe as possible so that they bind to the same specific or non-specific targets synchronously. Second, the differences of signal intensity between PM- and MM-target pairs should be as large as possible to allow for confident assignment of positive signals and elimination of false positives. To find a balance between the above two conflicting factors, two key design parameters, the number and distribution of mismatches, were examined in this study. Our results showed that choosing an appropriate number of mismatches for a MM probe depends on hybridization temperature (Figure 2). Theoretically, for short (25-mer) oligonucleotide probes, one mismatched nucleotide in the MM probe string is enough to obtain a high level of discrimination [29]. A single mismatch was also sufficient for detection of single nucleotide polymorphisms (SNP) [18]. However, since the relative signal of single mismatch probes was still around 0.60 for long (50-mer) oligonucleotides (Figure 2A), it is obvious that one mismatched nucleotide is insufficient to distinguish the PM signal from its MM signal. Therefore, in order to achieve sufficient discrimination, multiple mismatches are required. In addition, it is known that in solution the DNA duplex is more stable at lower temperatures for both specific and nonspecific binding. This phenomenon could be easily explained by the decrease of free energy, which would influence the performance of both MM and PM probes. However, we found that MM probes clearly acquired more signal than PM probes as the temperature decreased, suggesting that MM probes were more sensitive to temperature than PM probes. Therefore, three, four and five mismatch nucleotides are required for hybridization at 50°C, 45°C and 42°C, respectively, for MM oligonucleotide probes to achieve 10% discrimination.

The comparison of signals from randomly and evenly distributed MM probes clearly demonstrates that evenly distributed mismatches generally have a higher discrimination power than randomly distributed mismatches. This is consistent with all previous microarray studies using both long and short MM oligonucleotide probes [11,23]. However, when we examined the evenly distributed MM probes individually, there appeared to be a large variability in discrimination power among the different genes (Figure 6), suggesting that the even distribution strategy for MM probe design needs further improvement for individual genes. Therefore, a novel MPDNN model was constructed for this purpose. Since the influence of the chip surface on the hybridization behavior of probes is still unclear, most currently available probe design programs use nearest neighbor parameters, which are adapted from solution to microarray systems [30,41]. But recent experimental results have shown that the difference in thermodynamics between solution and microarray systems is considerable [23,42,43]. In microarray systems, probes are cross-linked and fixed on a solid surface (*e.g.*, glass slides), and the disequilibrium between probes and their targets is expected to be fundamentally different from the DNA duplex in solution. As a result, some thermodynamic parameters of dimer nucleotides which are equivalent in solution, such as CC-GG and GG-CC, AG-TC and TC-AG, become distinguishable. This phenomenon has been observed previously [20,23]. Moreover, there was a significant positional effect of mismatches in the microarray environment [20,23]. One of the consequences of these effects is that probes with evenly distributed mismatches have lower signal intensities than randomly distributed probes, contrary to the free energy calculations carried out based on solution chemistry. In the nearest neighbor calculation, the free energy of probes with the same number of mismatches should be similar regardless of where the mismatches are located, except for adjacent mismatches or those at both ends.

For the above reasons, the modification of thermodynamic parameters in microarray systems appears necessary. Zhang et al. (2003) built a PDNN model to correct these parameters and improve data analysis for gene expression. But the modifications were based only on data from Affymetrix^® ^arrays. Since the probe sets and array construction methods are different for spotted microarrays, we modified this model to fit our single-paired PM/MM probes for spotted arrays. The MPDNN model is based on a simple deduction that relative signal intensities and relative free energies are linearly dependent (Figure 3), which was supported by our regressive analyses that the linear relationship had higher relative coefficient (R = 0.798) than logarithmic (R = 0.736) or power (R = 0.741) correlations. Additionally, there are three more reasons for using the relative signal intensity in this study rather than the original signal intensity. First, the relative signal intensity is a normalized intensity with each PM signal set at 100% so that a gene-dependent factor may be eliminated. Second, the coefficient of variation (CV) of the relative signal intensities among replicate slides was 21% on average, less than the 29% obtained from the original signal intensities, indicating the relative signal intensities were more stable among the replicates. In addition, most microarray users focus only on relative signal intensities for relative comparisons rather than the original intensities. Thus, the linear relationship of relative signal intensities and relative free energies is suitable for mathematical modeling.

We used the new MPDNN model to generate a set of improved parameters for dimer matched and mismatched nucleotides and employed these parameters to redesign MM probes. The basic idea for the new design process is to control the relative signals for each individual gene by exchanging different nucleotides in probe strings (Figure 5). As a result, the redesigned MM probes had slightly flexible mismatch positions which were different from the commonly used fixed-position design methods. Experimental data demonstrated that the relative signal intensities from the redesigned MM probes were less variable than the fixed-position MM probes, indicating that the new design method with flexible mismatch positions was better than the previous method. Interestingly, the MM probes redesigned using MPDNN contained 16 G to A changes among the 40 total mismatches, much higher than other base changes. Some studies showed that the Δ*T*_m _of the G to A change was one of biggest among all base changes [44]. This implies that different nucleotide exchanges would affect signal intensities differently. Therefore, theoretically we could design MM probes for any length of probes using the MPDNN method.

Like other commonly used models [11,20], the MPDNN model is simply based on a linear relationship between signal intensity and free energy. Other factors were ignored in the final formula, such as target labeling efficiency and fluorescence on redundant targets. This model does have some limitations. For example, the adjusted thermodynamic parameters of mismatched dimer nucleotides through MPDNN are variable at different temperatures (data not shown). Also, hybridization at 50°C and 50% formamide is equivalent to 80°C, which is very close to some probe-target melting temperatures, resulting in more variation in thermodynamic parameters at 50°C than 42 or 45°C. This suggests that an optimization of microarray hybridization conditions is necessary to assure the quality of microarray data. Besides, we only studied nucleotide exchanges from As or Ts to Gs and Cs or Gs to As, and only DNA-DNA duplexes were tested. In RNA-DNA microarray hybridizations, the exchange from As to Gs will be GU wobble with a similar interaction energy to AU case. Therefore, more data from other exchanges are needed to improve MPDNN model in the future.

To our knowledge, this is the first report of MM probe design parameters that are experimentally established for long (50-mer) oligonucleotides, and this will provide general guidance for microarray MM probe design. The MPDNN model can be further validated by other researchers in both experimental and theoretical fields, and potentially integrated into probe design software like CommOligo (Li et al. 2005).

## Conclusion

MM probes must be carefully designed and evaluated before incorporation into both short and long oligonucleotide microarrays. This study provides guidance on the design of MM probes for long oligonucleotides (e.g., 50 mers). In general, the results demonstrated that the probes with evenly distributed mismatches were more distinguishable than those with randomly distributed mismatches. MM probes (50 mers) with 3, 4 and 5 mismatched nucleotides could be differentiated when hybridized at 50, 45 and 42°C, respectively. Additionally, instead of simply altering the nucleotides at fixed positions along the probe string, we constructed a model to calculate the relative free energy based on microarray data, and then used this model to further improve MM probe design. The results demonstrate that this novel MPDNN model can dramatically improve the consistency of long MM probe design. Also, this modeling method can potentially be used for the prediction of oligonucleotide hybridization on microarrays.

## Authors' contributions

YD, ZH and JZ designed these experiments. YD carried out all experiments and analyses and JV helped organize experiments and printed the microarrays. YD, ZH, JV and JZ wrote the manuscript together.

## Supplementary Material

Additional file 1 **The PM and MM probes in this study.** the column 1 shows the probe names and column 2 shows their values of relative signal intensities. The columns 3 and 4 show the sequences of probes and their paired sequences and column 5 shows the positions of mismatch nucleotides. Click here for file
